# Cortical thickness distinguishes between major depression and schizophrenia in adolescents

**DOI:** 10.1186/s12888-021-03373-1

**Published:** 2021-07-20

**Authors:** Zheyi Zhou, Kangcheng Wang, Jinxiang Tang, Dongtao Wei, Li Song, Yadong Peng, Yixiao Fu, Jiang Qiu

**Affiliations:** 1 Key Laboratory of Cognition and Personality (SWU), Ministry of Education, Chongqing, 400715 China; 2 Faculty of Psychology, Southwest University, No.2 Tiansheng Road, Beibei District, Chongqing, 400715 China; 3 Faculty of Psychology, Shandong Normal University, Jinan, 250014 Shandong China; 4 Department of Psychiatry, The First Affiliated Hospital of Chongqing Medical University, No.1, Yixueyuan Road, Yuzhong District, Chongqing, 400016 China; 5 Sleep and Psychology Center, The Bishan Hospital of Chongqing, Chongqing, 402760 China; 6 Department of Psychology, Chongqing Health Center for Women and Children, Chongqing, 401147 China; 7 Collaborative Innovation Center of Assessment Toward Basic Education Quality, Southwest University Branch, Beijing Normal University, Beijing, 100875 China

**Keywords:** Depression, Schizophrenia, Adolescence, Cortical thickness, Machine learning

## Abstract

**Background:**

Early diagnosis of adolescent psychiatric disorder is crucial for early intervention. However, there is extensive comorbidity between affective and psychotic disorders, which increases the difficulty of precise diagnoses among adolescents.

**Methods:**

We obtained structural magnetic resonance imaging scans from 150 adolescents, including 67 and 47 patients with major depressive disorder (MDD) and schizophrenia (SCZ), as well as 34 healthy controls (HC) to explore whether psychiatric disorders could be identified using a machine learning technique. Specifically, we used the support vector machine and the leave-one-out cross-validation method to distinguish among adolescents with MDD and SCZ and healthy controls.

**Results:**

We found that cortical thickness was a classification feature of a) MDD and HC with 79.21% accuracy where the temporal pole had the highest weight; b) SCZ and HC with 69.88% accuracy where the left superior temporal sulcus had the highest weight. Notably, adolescents with MDD and SCZ could be classified with 62.93% accuracy where the right pars triangularis had the highest weight.

**Conclusions:**

Our findings suggest that cortical thickness may be a critical biological feature in the diagnosis of adolescent psychiatric disorders. These findings might be helpful to establish an early prediction model for adolescents to better diagnose psychiatric disorders.

**Supplementary Information:**

The online version contains supplementary material available at 10.1186/s12888-021-03373-1.

## Background

Psychiatric disorder is among the most important causes of mortality in humans, which affects the quality of life and increases the social burden [1–3]. Psychotic (such as schizophrenia [SCZ]) and affective disorders (such as major depressive disorder [MDD]), as two typical psychiatric disorders, have extensive comorbidities with each other [4, 5]. Approximately 80% of patients with SCZ experience depressive episode in the early disorder stages [6]. The depression prevalence among patients with SCZ can be as high as 40% [7, 8]. Moreover, patients with MDD have been shown to have a higher risk of developing a psychotic disorder. In addition, depression often precedes psychotic symptoms in people with a high risk of SCZ [9, 10]. The presence of psychotic symptoms in patients with depression is considered a clinical depression subtype known as psychotic depression, which is associated with increased depressive symptom severity [11, 12]. Further, both MDD and SCZ significantly impair working memory, planning, shifting and so on [13, 14]. In addition, MDD and SCZ have significant genetic similarities [15, 16]. This complex relationship between MDD and SCZ sometimes can impede diagnoses by psychiatrists.

Adolescence is the critical period during psychological development in which most psychiatric disorders are initially detected [17, 18]. Similarly, there is an overlap in the clinical characteristics of adolescents with MDD and SCZ [19]. At this stage, although there are not wide effects on behaviors influenced by psychiatric disorders, they can have significant negative effects later in life and are potential health threat for future generations [20, 21]. Prior to severe symptoms during adulthood, SCZ often begins developing during early adolescence [18, 22, 23]. Compared to patients with adult-onset SCZ, those with early-onset often exhibit more severe psychotic symptoms, poorer therapeutic outcomes, and greater disability [24, 25]. In structural neuroimaging studies, although a meta-analysis by Van et al. found a thinner cortex in adult patients with SCZ (especially in the frontal and temporal lobe regions), Thormodsen et al. reported no significant difference in the cortical thickness between adolescent patients with SCZ and healthy adolescents [26, 27]. These findings indicate changes in cerebral cortex of the adolescent patients with SCZ may take time to develop.

The first episodes of affective disorder, including MDD, appear at adolescence and cause serious distress to the patients and their guardians [28, 29]. In the symptoms of MDD, appetite and weight changes, energy loss, and insomnia can be seen among adolescents while concentration problems and anhedonia/loss of interest are more frequent among adults [30]. Structural neuroimaging studies have reported reduced cortical thickness in the dorsal lateral prefrontal cortex, lingual gyrus, and pre- and postcentral gyrus in patients with early-onset depression [31]. Reynolds et al. reported a thicker bilateral dorsal-lateral prefrontal cortex and left caudal anterior cingulate cortex in MDD adolescents [32]. Contrastingly, previous studies have reported no significant differences in the cortical thickness at the whole-brain level between adult patients with MDD and healthy controls [31, 33]. Consistent with findings on SCZ, these findings indicate there are a lot of differences in symptoms and brain morphology between adolescents and adults with depression. Therefore, pathophysiological mechanisms might differ between adolescents and adults with MDD.

Machine learning is an emerging technology in recent years that can help us better understand the pathophysiological mechanisms of the brain. It involves assessing the similarity of a brain MRI scan with images obtained from a group of individuals to determine whether the tissue is more likely from a patient or a healthy individual [34]. Over the past decade, different machine learning methods using various brain features have been developed to distinguish between psychotic and affective disorders with a good accuracy ranging from 60 to 90% [35–38]. However, these studies on disease identification have mostly focused on adults without accounting for pathophysiology differences at different stages, especially in adolescence, which is a critical development period. It remains unclear whether adolescent patients with MDD and SCZ can be distinguished via structural brain MRI scans.

Consequently, we used a support vector machine (SVM) to determine whether it could be used to accurately identify adolescent patients with SCZ and MDD at the individual level based on anatomic brain parameters, as well as to determine their key brain characteristics [39]. We hypothesized that SVM could accurately distinguish among MDD, SCZ, and healthy controls. To our knowledge, this is the first study to examine psychiatric disorders (MDD and SCZ) in adolescents using a machine learning technique. This study provides the insight of MDD and SCZ. Moreover, this study may contribute toward the identification of adolescent psychiatric disorders based on MRI scans and a scientific basis for early clinical diagnosis of psychiatric disorder.

## Methods

### Participants

The patients were diagnosed using a Structured Clinical Interview for Diagnostic and Statistical Manual of Mental Disorders (SCID-I/P, Chinese version) by two psychiatrists in the Department of Psychiatry, The First Affiliated Hospital of Chongqing Medical University between July 2015 and October 2017 [40]. All patients were screened for comorbidities of depression and schizophrenia to ensure that every patient had only one of the disorders at the time of diagnosis. The initial sample comprised 175 participants, including 80 patients with MDD, 61 patients with SCZ, and 34 age- and gender-matched HC. We excluded participants aged < 10 years (*N* = 1) and > 20 years (*N* = 2). Moreover, we excluded 22 participants due to identified head motion artifacts after two specialists visually inspected the original and segmentation images. Finally, we included 150 participants, including 67 patients with MDD, 49 patients with SCZ, and 34 HC. We assessed the history of diseases for all participants to exclude any existing systemic diseases, including neurologic diseases and morphologic anomalies in the brain.

This study was approved by the Local Medical Ethics Committee of the First Affiliated Hospital of Chongqing Medical University. All methods were performed in accordance with the relevant guidelines and regulations. All the study participants provided written assent and their legal guardians provided written informed consent.

### Magnetic resonance imaging data acquisition

All the participants were scanned on a 3 Tesla GE Signa Medical Systems (Milwaukee, Wisconsin, USA) with a 12-channel head coil at The First Affiliated Hospital of Chongqing Medical University. We acquired high-resolution anatomical T1-weighted spoiled gradient-recalled images covering the whole brain (TR = 8348 ms, TE = 3272 ms, 156 axial slices, flip angle = 12°, field of view = 240.128 × 240.128 × 156 mm, matrix = 512 × 512, voxel size = 0.469 × 0.469 × 1 *mm*^3^).

### Magnetic resonance imaging data preprocessing

The T1-weighted structural scans were processed using FreeSurfer (version 5.3.0, http://surfer.nmr.harvard.edu) image analysis suite to produce measures of gray matter thickness [41, 42]. Using an automated brain segmentation process, the command “recon-all” was executed to estimate the brain region volume based on the Desikan-Killiany atlas [43]. Both original and processed images were visually inspected by two specialists to identify excessive motion artifacts. According to the proposal of Klapwijk et al., the criteria for visual quality control in this study include: (1) whether the reconstructed image is affected by movement; (2) whether the temporal pole is missing in the reconstruction; (3) whether the non-brain tissue is included in the reconstruction of the pial surface; (4) whether parts of the cortex are missing in the reconstruction [44]. A 4-point score was used. If either of the two specialists thought the result of any above item was bad (score 1), this participant would be excluded. The Euler number of all images in this study are 2, which indicates the high data quality for cortical reconstruction. Moreover, no manual corrections were applied. After the execution of the command “recon-all” which contains a series of automatic preprocessing steps such as Talairach transform computation and spherical registration [45, 46], the entire cortical surface was parcellated into 34 regions per hemisphere [47]. Given the reported cortical thickness abnormalities of MDD and SCZ and the evidence that there are less individual variations in cortical thickness than in cortical gray matter volume, we used cortical thickness as the main index [48–50]. However, other brain structure aspects (e.g., cortical and subcortical volume, cortical surface area) provide information toward a broader understanding of these disorders; therefore, we investigated these parameters as secondary indices.

### Statistical analysis

SVM is a type of multivariate classification algorithm automatically identifying the hyperplane that differentiates two labeled classes in a training data feature set. Subsequently, the individual (test data set) is automatically classified or predicted by the hyperplane. This method is suitable for high-dimension imaging data set analysis [39]. As a diagnostic tool, SVM has been applied to MRI data to predict various pathologies, including MDD, SCZ, bipolar depression, etc. [35, 36, 38, 51, 52]. We performed statistical analysis using the Library for Support Vector Machines (LIBSVM) software package and Matlab 2017b (www.mathworks.com) [53]. The cortical gray matter thickness of 68 brain regions was selected as the model features without a priori regions of interest. To remove the influence of sex, age and intracranial volumes (ICV) while retaining disease-associated neuroanatomical variations, we regressed the original data to correct for sex, age and ICV effects [54]. Subsequently, to avoid the effect of differences in the magnitude of cortical thickness across brain regions on the weight values, we standardized the regressed data through Z-transformation. To better explore the effect of different brain regions on classification for future clinical application, we build three models: (1) separating MDD from HC; (2) separating SCZ from HC; (3) separating MDD from SCZ. Each model was generated by C-SVC with a linear kernel due to the high dimensionality of the data [52]. Participants in each model were classified using two nested leave-one-out cross-validations (LOO-CV). Here, one participant is excluded as the testing set while the remaining participants are used as the training set within each iteration. To identify the best classifier parameter, we performed a search over parameter C, a cost parameter of SVM classifier, whose values were in the set [*C* = 2^−3^, 2^−2^, 2^−1^, …, 2^3^]. For each value of *C*, the accuracy rate was measured using another leave-one-out cross-validation within the training set. The *C* parameter that produced the greatest classification effect in the training set was computed by the model. After identifying the best parameter, the left-out testing set was classified to determine the classification rate of the model. To further enrich the study, we also computed balanced accuracy (BA) with the posterior probability interval (PI) of our model because of the unbalanced number between groups [55]. Finally, the accuracy of all three models was confirmed through permutation tests separately [56]. We randomized the labels (i.e., group membership as MDD or SCZ) with a held constant ratio of 3000 times and calculated the classification accuracy within each iteration. *P*-values, the number of times that the accuracy was higher than our original classification accuracy divided by the iteration times, reflected the significance of our classification models.

## Results

### Sample characteristics

No evidence of a group difference was found in the variables of gender (MDD: 42% male; SCZ: 49% male; and HC: 44% male) and age (MDD: mean age = 16.22 ± 2.02 years; SCZ: 16.02 ± 1.80 years; HC: 16.32 ± 2.99 years). moreover, there was no significant among-group difference in the intracranial volume. There was no significant difference in the current episode duration, age at onset between the MDD and SCZ groups. Compared to the number of patients with MDD, More patients with SCZ undergo medication and physical therapy, including electric shock and transcranial magnetic stimulation, which is consistent with their pathology [57]. Table 1 presents the clinical and demographic characteristics, as well as their between-group comparisons.

**Table 1:** Clinical and demographic characteristics

Measures	MDD (*N* = 67)	SCZ (*N* = 49)	HC (*N* = 34)	*p*-value
Age (year)	16.22 ± 2.02	16.02 ± 1.80	16.32 ± 2.99	0.81^a^
Intracranial Volume	1459.73 ± 127.77	1448.33 ± 141.57	1484.68 ± 108.24	0.44^a^
Length of Current Episode (months)	7.88 ± 9.19	6.24 ± 12.29	–	0.41^b^
Age at Onset (year)	15.13 ± 2.17	15.39 ± 2.10	–	0.53^b^
Male (%)	28 (41.79)	24 (48.98)	15 (44.12)	0.56^c^
**Prior Exposure to Medicine (%)**	**34 (50.75)**	**42 (85.71)**	**–**	**< 0.0001**^d^
First Episode (%)	48 (71.64)	42 (85.71)	–	0.11^d^
Family History of Mental Disorders (%)	7 (10.45)	8 (16.33)	–	0.41^d^
**Physical Intervention (%)**	**12 (17.91)**	**27 (55.10)**	**–**	**< 0.0001**^d^

Values indicate the mean ± SD

Abbreviations: *MDD* Major Depressive Disorder, *SCZ* schizophrenia, *HC* healthy controls

^a^Statistic computed using F-test

^b^Statistic computed using two-sample t-tests

^c^Statistic computed using *χ*^2^ test

^d^Statistic computed using Fisher’s exact test

### SVM classification

**Fig. 1:**
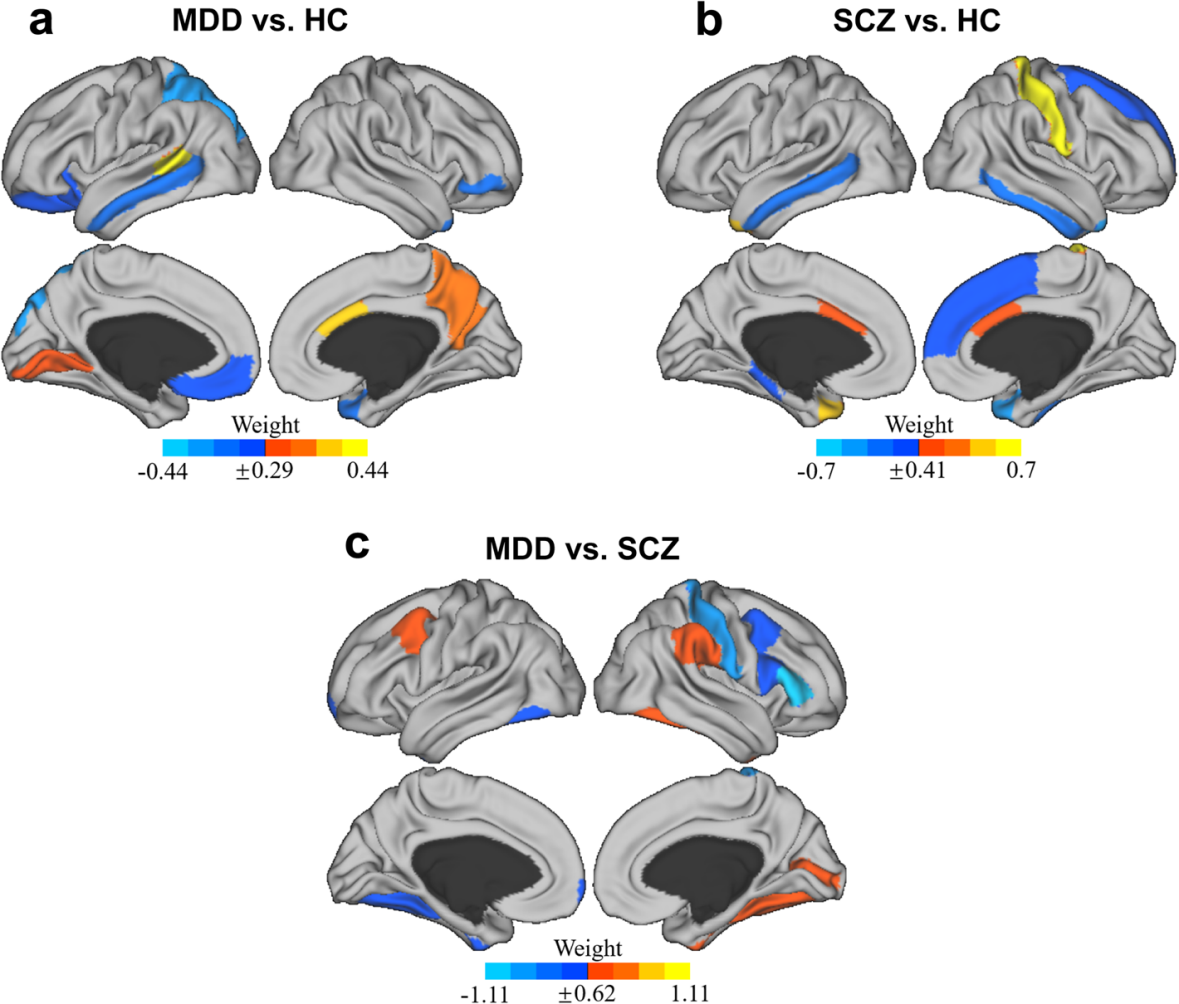
The top 10 thickness brain regions contributing to classification accuracy in the SVM. **a** Brain regions with their thickness having the highest weight to distinguish patients with major depression and healthy controls. **b** Brain regions with their thickness having the highest weight to distinguish patients with schizophrenia and healthy controls. **c** Brain regions with their thickness having the highest weight to distinguish patients with major depression and schizophrenia

In case-classification, distinguishing patients with MDD (positive class) and SCZ (positive class) from HC using cortical gray matter thickness resulted in an accuracy of 79.21% (*p* = .002, 95% CIs of permutation test(per_CIs): 39.60–71.29%, sensitivity: 83.58%, specificity: 70.59%, BA: 76.20% (95% PI: 66.72–83.88%)) and 69.88% (*p* = .008, 95% per_CIs: 38.55–66.27%, sensitivity: 73.47%, specificity: 64.71%, BA: 68.45% (95% PI: 58.02–77.52%)), respectively. The model of MDD-SCZ (MDD as the positive class) resulted in an accuracy of 62.93% (*p* = .045, 95% per_CIs: 37.07–64.66%, sensitivity: 64.18%, specificity: 61.22%, BA: 62.25% (95% PI: 53.38–70.48%)), which was lower than the case-classification. Figure 1 present the top 10 averaged weights of the brain regions in each classification model. Specifically, the right postcentral gyrus, the left temporal pole, and the right temporal pole were the most important brain regions for MDD-HC classification. On the other hand, the left bank superior temporal sulcus, left superior parietal gyrus, and right caudal anterior cingulate cortex were the most important brain regions for SCZ-HC classification. Regarding MDD-SCZ classification, the heavy-weighted regions were distributed across different brain regions. The right pars triangularis, right postcentral gyrus, and caudal middle frontal gyrus were the most important for MDD-SCZ classification.

Further, we explored the effect of the cerebellar-subcortical volume, gray matter volume, and gray matter area on classification, respectively. An additional table presents the key model information and classification results [see Additional file 1]. Compared to the results using cortical thickness as the feature set, all the results using other brain index as feature set were worse in all classification models, except using cerebellar-subcortical volume as the feature set alone to distinguish MDD and SCZ, whose accuracy (62.93%) was the same with it using cortical thickness.

## Discussion

To our knowledge, this is the first MRI study to distinguish between adolescent psychiatric disorders (MDD and SCZ) using machine learning techniques. We employed a linear kernel nested SVM to create data-driven models for classifying patients with MDD, patients with SCZ, and HC based on whole-brain neuroanatomical features in MRI scans. The models using cortical thickness could distinguish adolescents with MDD and SCZ from healthy adolescents with an accuracy of 79.21, and 69.88%, respectively. The classification between adolescents with MDD and those with SCZ had a lower accuracy of 62.93%. Our findings indicate that machine learning using cortical thickness as the features can allow effective classification of psychiatric disorders among adolescents at an individual level.

Our findings indicate that structural brain MRI imaging can be used to effectively identify MDD and SCZ in adolescents. Previous studies widely used different structural indexes, including subcortical volume, gray matter density, gray matter volume, cortical thickness, and cortical area, and all of them could distinguish between adults with psychiatric disorders and healthy adults [38, 58–60]. In this study, we focused on adolescent psychiatric patients and collected structural MRI data from patients with MDD and SCZ. Different structural indexes were used as SVM model features respectively for case- classification and MDD-SCZ classification. Unlike previous findings on adults, only cortical thickness could provide the best accuracy in three adolescent classification models in our study. This is consistent with the findings by Qiu et al., who used an SVM based on various brain morphometric features to distinguish between 32 adult patients with first-episode MDD and 32 HC [61]. They reported that multiple cortical features could discriminate them with cortical thickness providing the highest accuracy. Our findings indicate that the cortical thickness is already altered in adolescent patients with MDD and SCZ. This is consistent with previous findings that patients with childhood-onset schizophrenia presented with bilateral deficits in the temporal, prefrontal, and parietal cortices [62]. Moreover, using the machine learning technique, cortical thickness has been reported to predict future-onset of depression in adolescents with an accuracy of 70% [63]. Besides, with volumes of both subcortical and cerebellar regions as the feature set, the classification model of MDD-SCZ resulted in a significant accuracy of 62.93% (*p* = .044). This is because some subcortical nuclei are also linked to MDD and SCZ, such as amygdala, which is associated with emotion [64, 65]. In addition, there is an interesting finding that we succeed to distinguish adolescents with SCZ and HC but Thormodsen et al. not [26]. In their study., there are no significant evidence of cortical thickness difference between adolescent with SCZ and HC based on univariate analysis. In our study, to further explore the brain morphology of adolescents with SCZ, we succeed to distinguish them using multivariate analysis. Although no significant evidence is found in cortical thickness of each brain region between the two groups, there may be a particular spatial pattern of abnormal changes in cortical thickness across brain regions in adolescents with SCZ. That may be why we are successful. In a word, cortical thickness is a crucial structural brain index for identifying adolescent patients with psychiatric disorders.

For distinguishing adolescent patients with MDD from HC, the most important brain region was the temporal pole. The temporal pole, which is a node of the paralimbic system, plays an important role in socioemotional and cognitive processing [66]. Defects in these processes are associated with depression [67, 68]. Gray matter abnormities in the temporal pole have been reported in medication-naive patients with first-episode MDD [69, 70]. Compared to healthy controls, individuals with depression present with greater activation of the right anterior temporal pole [71]. Previous studies also reported abnormal functional connections between the right temporal pole and other brain regions in patients with MDD [72–74]. Given the emotional instability in adolescents and the abnormal emotional response to external stimuli, abnormal changes are more likely to occur in the temporal pole [75]. Therefore, the structure of this region could be used as a crucial biomarker for adolescent depression.

The left banks of the superior temporal sulcus, which is a crucial association area for biological motion perception, was the most significant brain region for distinguishing between adolescents with SCZ and HC [76]. The superior temporal sulcus is part of a neural circuit involved in perceiving intention from action and reactions to social and emotional events [77, 78]. Many studies have reported a reduced ability to extract social information from bodily cues in patients with SCZ [79–82]. Neuroimaging studies have reported that patients with SCZ present with an aberrant pattern of superior temporal sulcus activity during basic biological motion tasks [83, 84]. Matsumoto et al. reported a negative correlation of the behavioral performance on basic biological motion perception tasks and the gray matter volume of the superior temporal sulcus in patients with SCZ [85]. Similarly, we observed adolescents with SCZ had thinning cortical thickness of the left banks of the superior temporal sulcus than HC (*p* = 0.005, FDR corrected). Our findings indicate that the superior temporal sulcus could be associated with impaired extraction of social information in adolescents with SCZ.

In our study, the most important brain region that distinguishing between MDD and SCZ was the right pars triangularis. The pars triangular is located in the inferior frontal gyrus, which is a crucial brain region for emotional and cognitive control circuits [86]. Deng et al. reported that the right inferior frontal gyrus is highly activated in a stop-signal task involving motor inhibitory responses [87]. Damage to this area impairs the performance of the stop-signal task [88]. Moreover, individuals with higher depression levels were found to have poorer response inhibition and to perform worse on the stop-signal task [89]. Neuroimaging studies have reported that patients with MDD have increased functional connectivity in the right pars triangularis of the inferior frontal gyrus [90, 91]. This indicates a strong correlation of the right pars triangularis with depression in adolescents. Patients with SCZ also present with reduced gray matter volume in the right inferior frontal gyrus [92]. However, this is attributed to the generalized neuropsychological impairment associated with SCZ rather than impaired inhibitory behavioral control, which is a specific cognitive impairment [93]. Taken together, these findings indicated that the right pars triangularis is associated with response inhibition in adolescents with MDD and could be used to distinguish between adolescent patients with MDD and SCZ.

This study has several limitations. First, the accuracies of our models were all < 80%. To improve accuracy, we combined other indexes (cortical volume, cortical area, and cerebellar-subcortical volume) with cortical thickness as the feature set. An additional table presents these results [see Additional file 2]. After adding additional indexes into the feature set, no improved prediction accuracy was found. To prevent model overfitting and to improve accuracy, we applied the least absolute shrinkage and selection operator for feature selection and dimensionality reduction [94]. However, this did not reduce dimensions, which could be attributed to the complexity of brain structures and small sample size. In addition, it is a limitation that we exclude those patients with psychiatric comorbidities to maximize the group difference to train the classifier. The patients with comorbidities are valuable cases to investigate for diagnostic purposes. In the future, we will apply the models here to these groups. Moreover, we obtained our sample from a single center. It is not clear whether our results are reproducible and generalizable. In future studies, we will obtain multi-center samples to validate these findings and continue to focus on early psychiatric disorders.

## Conclusions

In summary, using a machine learning technique, we found that cortical thickness contributed toward distinguishing adolescent patients with MDD and SCZ. This indicates that there are early-life structural brain abnormalities in patients with MDD and SCZ. These findings contribute toward biomarker-based clinical diagnosis and demonstrate the utility of pattern recognition in exploring the neurological basis of psychiatric disorders. Further, this study provides an evidence regarding the correct identification of adolescent psychiatric disorders based on neuroimaging. Future studies will focus on identifying other psychiatric disorders to improve the identification accuracy of specific diseases to contribute to early diagnosis and treatment of psychiatric diseases.

## Supplementary Information

**Additional file 1: Supplementary Table 1.** Comparison of the model classification accuracy of the different brain indexes.

**Additional file 2: Supplementary Table 2.** Comparison of the model classification accuracy of gray matter thickness with the different brain indexes.

**Additional file 3: Supplementary Table 3.** The processed data supporting the conclusions of this article (cortical thickness).

**Additional file 4: Supplementary Table 4.** The mean feature weight and cross-validation ratio of all brain regions.

**Additional file 5: Supplementary Table 5.** The t-value and *p*-value between different groups using t-test in univariate analysis (FDR corrected).

## Data Availability

The datasets analysed during the current study are available in Additional file 3*.* The code analysed during the current study is available in https://github.com/zhouzheyi/adolescent_psychiatry_svm.

## Abbreviations

MDD: Major depressive disorder
SCZ: Schizophrenia
HC: Healthy controls
MRI: Magnetic resonance imaging
SVM: Support vector machine
BA: Balanced accuracy
FDR: False discovery rate
PI: Posterior probability interval
CI: Confidence interval
per_CIs: Confidence intervals of permutation test
