# Supplementary material for: Cortical thickness distinguishes between major depression and schizophrenia in adolescents
Source: BMC Psychiatry. 2021 Jul 20;21:361. doi: 10.1186/s12888-021-03373-1 (PMC8293570; doi:10.1186/s12888-021-03373-1)
Supplement: Supplementary file 1 — Additional file 1: Supplementary Table 1. Comparison of the model classification accuracy of the different brain indexes. [file 12888_2021_3373_MOESM1_ESM.docx]

Supplementary Table 1. Comparison of the model classification accuracy of the different brain indexes

| Measures | Features number | MDD vs. HC (N = 101) | SCZ vs. HC (N = 83) | MDD vs. SCZ (N = 116) |
| --- | --- | --- | --- | --- |
| Cerebellar-subcortical volume | 38 | 69.31% | 66.27% | 62.93% |
| Gray matter volume | 68 | 48.51% | 53.01% | 61.21% |
| Gray matter area | 68 | 61.39% | 50.60% | 52.59% |

Note. MDD, Major Depressive Disorder; SCZ, schizophrenia; HC, healthy controls.
