# Supplementary material for: Cortical thickness distinguishes between major depression and schizophrenia in adolescents
Source: BMC Psychiatry. 2021 Jul 20;21:361. doi: 10.1186/s12888-021-03373-1 (PMC8293570; doi:10.1186/s12888-021-03373-1)
Supplement: Supplementary file 2 — Additional file 2: Supplementary Table 2. Comparison of the model classification accuracy of gray matter thickness with the different brain indexes. [file 12888_2021_3373_MOESM2_ESM.docx]

Supplementary Table 2. Comparison of the model classification accuracy of gray matter thickness with the different brain indexes

| Measures | Features number | MDD vs. HC (N=101) | SCZ vs. HC (N=83) | MDD vs. SCZ (N=116) |
| --- | --- | --- | --- | --- |
| Gray matter thickness & cerebellar-subcortical volume | 106 | 77.23% | 62.65% | 56.03% |
| Gray matter thickness & volume | 136 | 72.28% | 61.45% | 55.17% |
| Gray matter thickness & area | 136 | 68.32% | 53.01% | 47.41% |

Note. MDD, Major Depressive Disorder; SCZ, schizophrenia; HC, healthy controls.
